# Refining the Phenotype of Recurrent Rearrangements of Chromosome 16

**DOI:** 10.3390/ijms20051095

**Published:** 2019-03-04

**Authors:** Serena Redaelli, Silvia Maitz, Francesca Crosti, Elena Sala, Nicoletta Villa, Luigina Spaccini, Angelo Selicorni, Miriam Rigoldi, Donatella Conconi, Leda Dalprà, Gaia Roversi, Angela Bentivegna

**Affiliations:** 1 School of Medicine and Surgery, University of Milano-Bicocca, 20900 Monza, Italy; serena.redaelli@unimib.it (S.R.); donatella.conconi@unimib.it (D.C.); leda.dalpra@unimib.it (L.D.); gaia.roversi@unimib.it (G.R.); 2 Clinical Pediatric Genetics Unit, Pediatrics Clinics, MBBM Foundation, S. Gerardo Hospital, 20900 Monza, Italy; maitz.silvia@gmail.com; 3 Medical Genetics Laboratory, Clinical Pathology Department, S. Gerardo Hospital, 20900 Monza, Italy; f.crosti@asst-monza.it (F.C.); elena.sala@asst-monza.it (E.S.); n.villa@asst-monza.it (N.V.); rigoldimiriam@gmail.com (M.R.); 4 Clinical Genetics Unit, Department of Obstetrics and Gynecology, V. Buzzi Children’s Hospital, University of Milan, 20154 Milan, Italy; luigina.spaccini@asst-fbf-sacco.it; 5 Clinical Pediatric Unit, ASST Lariana, 22042 Como, Italy; angelo.selicorni61@gmail.com; 6 NeuroMI, Milan center of Neuroscience, University of Milano-Bicocca, Dept. of Neurology and Neuroscience, San Gerardo Hospital, 20900 Monza, Italy

**Keywords:** chromosome 16, array-CGH, CNV, developmental disability, speech disorder, two-hit model, 16p13.11 deletions and duplications, 16p11.2 deletions and duplications

## Abstract

Chromosome 16 is one of the most gene-rich chromosomes of our genome, and 10% of its sequence consists of segmental duplications, which give instability and predisposition to rearrangement by the recurrent mechanism of non-allelic homologous recombination. Microarray technologies have allowed for the analysis of copy number variations (CNVs) that can contribute to the risk of developing complex diseases. By array comparative genomic hybridization (CGH) screening of 1476 patients, we detected 27 cases with CNVs on chromosome 16. We identified four smallest regions of overlapping (SROs): one at 16p13.11 was found in seven patients; one at 16p12.2 was found in four patients; two close SROs at 16p11.2 were found in twelve patients; finally, six patients were found with atypical rearrangements. Although phenotypic variability was observed, we identified a male bias for Childhood Apraxia of Speech associated to 16p11.2 microdeletions. We also reported an elevated frequency of second-site genomic alterations, supporting the model of the second hit to explain the clinical variability associated with CNV syndromes. Our goal was to contribute to the building of a chromosome 16 disease-map based on disease susceptibility regions. The role of the CNVs of chromosome 16 was increasingly made clear in the determination of developmental delay. We also found that in some cases a second-site CNV could explain the phenotypic heterogeneity by a simple additive effect or a pejorative synergistic effect.

## 1. Introduction

Human chromosome 16 is a metacentric small-size chromosome belonging to the E group that spans 90.4 Mb with large numbers of repeats and variants localized mostly to the centromeric heterochromatin [1]. In a recent study using matrix algebra, Fatakia and colleagues unified intrinsic chromosomal parameters to derive an extrinsic effective gene density matrix, which constrains the non-random chromosomal arrangement in human interphase nuclei [2]. These data confirm that chromosome 16 is among the six most gene-rich chromosomes of our genome, despite the exact total number of genes being still unknown, probably due to the presence of a large number of pseudogenes. There are 2260 genes according to Vega Genome Browser release 68–2017 (http://vega.archive.ensembl.org/Homo_sapiens/Location/Chromosome?r=16) and 2006 genes according to NCBI, National Center for Biotechnology Information, Annotation Release 109–2018, (https://www.ncbi.nlm.nih.gov/gene?LinkName=nuccore_gene&from_uid=568815582), while there are 463 pseudogenes according to Vega Genome Browser release 68–2017. As a matter of fact, chromosome 16 contains several immunoglobin-like paralogues—a type of gene for which it is clearly evolutionarily adaptive to undergo rapid duplication followed by random mutation, as they play a role in adapting to potential antigens. It is interesting to note that 9.89% of chromosome 16 consists of segmental duplications (≥90% sequence identity and ≥1 kb in length), and a similar percentage corresponds to the unsequenced DNA of this chromosome [3]. In the “Guide to the Human Genome” (http://www.cshlp.org/ghg5_all/section/dna.shtml), 12.7% of chromosome 16 was reported as unsequenced. Intrachromosomal duplications are longer and have higher sequence identity than interchromosomal duplications, the former type shows greater than 97% sequence identity, consistent with a recent expansion 30–40 million years ago [4], although data on more recent duplications are emerging [5]. As a result, this complex architecture leads to strong instability and a predisposition to a recurrent mechanism of rearrangement via non-allelic homologous recombination (NAHR) between neighboring intrachromosomal segmental duplications. Recently, advancements in chromosomal microarray technologies have allowed for the analysis of copy number variations (CNVs) that promote genetic variability in humans, but can also contribute to Mendelian [6] and complex disease risk. In this context, the clinical association between the 593 Kb proximal deletions and duplications at 16p11.2 and Neurodevelopmental Disorders (NDs) is well known [7], even if the deletions and duplications are also observed in normal individuals [8]. Several studies on the 16p11.2 CNVs showed the existence of partially mirroring phenotypes; the deletion is associated with autism spectrum disorders, intellectual disability (ID), behavioral disorders, congenital abnormalities (CAs), diabetes-independent obesity, and macrocephaly [9,10,11,12,13]. Interestingly, chronic activation of GABA_B_ receptors improved performance on a series of cognitive and social tasks known to be impaired in two different 16p11.2 deletion mouse models [14]. Conversely, the reciprocal duplication is associated with autism, schizophrenia, anorexia, and microcephaly [15,16,17]. According to a recent meta-analysis, there is a fourteen fold-increased risk of psychosis and a sixteen-fold increased risk of schizophrenia in individuals with micro-duplication at the proximal 16p11.2 [18]. More recently, Raca and colleagues extended the phenotype of the ~550 kb 16p11.2 recurrent microdeletion syndrome to include a severe pediatric speech sound disorder (CAS: Childhood Apraxia of Speech) [19]. Another well-known critical region is the 1.5 Mb at 16p13.11. Deletions at this region are associated to ID, microcephaly, and epilepsy, while patients with the reciprocal duplication have, in addition to ID and/or CAs, conspicuous behavioral problems [20]. It is important to note that despite phenotypic variability, deletions inherited from clinically normal parents are likely to be causal for the patients’ phenotype, while the role of duplications (de novo or inherited) in the clinical phenotype is uncertain and may be a rare benign variant [20]. Finally, in a recent work, recurrent chromosome 16p13.1 duplications were reported as a risk factor for Thoracic Aortic Aneurysm and Dissection [21]. One possible explanation for variable expressivity in these genomic alterations emerges from a recent study that proposed a “two-hit” model in which the compound effect of a relatively small number of rare variants of large effect contributes to the phenotypic heterogeneity of genomic disorders [22].

In this work a diagnostic assessment by array comparative genomic hybridization (CGH) of 1476 patients (366 prenatal and 1110 postnatal), allowed us to identify 27 cases with CNVs on chromosome 16 (~1.8%). We contributed to the building of a chromosome 16 disease-map based on disease susceptibility regions with genotype–phenotype correlation attempts.

## 2. Results

**Figure 1:**
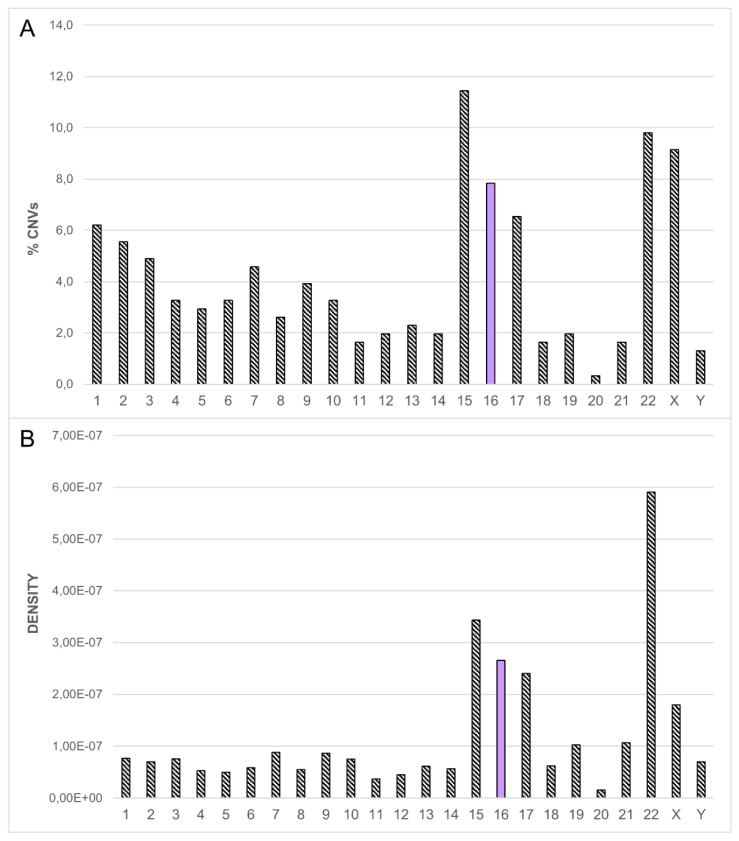
(**A**) Chromosomal distribution of pathogenetic and uncertain/unknown copy number variations (CNVs) in our patients (**B**) CNV density: number of CNVs in our patients in relation to the length of the chromosome. The purple column identifies chromosome 16.

In this work, we performed array CGH on 1476 patients (366 prenatal and 1110 postnatal diagnosis); the most frequent indications among postnatal cases were unexplained mental retardation and/or multiple congenital anomalies (MR/MRC). We identified a pathogenetic CNV in 167 cases (detection rate ~15%) and an uncertain/unknown CNV in 124 cases (detection rate ~11%). Our analysis showed that chromosome 16 follows chromosomes 15, 22, and X for chromosomal distribution of pathogenetic and uncertain/unknown CNVs in patients with apparently normal karyotype (Figure 1A). Furthermore, chromosome 16 follows chromosome 22 and 15 considering the CNV density (i.e., the number of CNVs in relation to the length of the chromosome) (Figure 1B). The first two chromosomes were expected, as the high density and clustering of CNVs fall into well-known regions, rich in segmental duplications involved in the recurrence of pathogenesis of NDs such as Prader-Willi/Angelman syndrome and DiGeorge/velocardiofacial syndrome, to name only the best known. Therefore, we decided to focus on the third chromosome.

We detected 27 cases with CNVs on chromosome 16 (16 males and 11 females) on a survey of 1476 participants, ~1.8%, one of the highest rates cited in the literature [6,16]. Table 1 provides patient information and the size and characteristics of each CNV.

**Figure 2:**
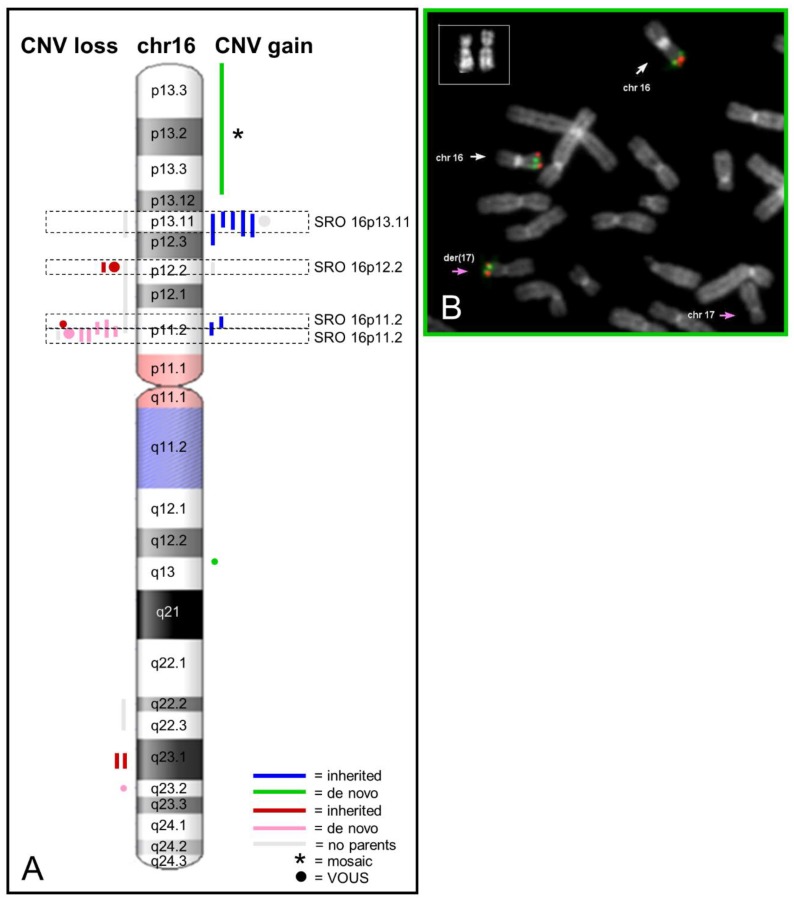
(**A**) Distribution of CNVs among chromosome 16. The dashed boxes show the smallest regions of overlapping (SROs). (**B**) FISH analysis on peripheral blood metaphases of patient 15 with 16p13.3 (red signals) and 16p13.13 (green signals) probes confirmed trisomic 16p13.1–16p13.3 regions. White arrows indicate chromosomes 16 and pink arrows chromosomes 17 (one normal and one with the two signals). 100× magnification.

We identified four common regions or SROs (smallest region of overlapping) (Figure 2): one at 16p13.11 included CNVs in seven patients; one at 16p12.2 was found in four patients; two close SROs at 16p11.2 were found in twelve patients. Finally, six patients were found with atypical rearrangements of chromosome 16.

Moreover, in order to unveil the disease-causing genes within each SRO, we applied an evolutionary genetic approach through the mapping of ohnologs. Ohnologs are genes retained after ancestral whole genome duplication events, which are inferred to be dosage-sensitive genes and probably responsible for a deleterious phenotype. We identified a total of 30 ohnologs, 24 of which were in a region covering 1.5 Mb in 16p11.2 (Supplementary Table S1).

### 2.1. Recurrent Microdeletion/Duplication of 16p13.11

**Figure 3:**
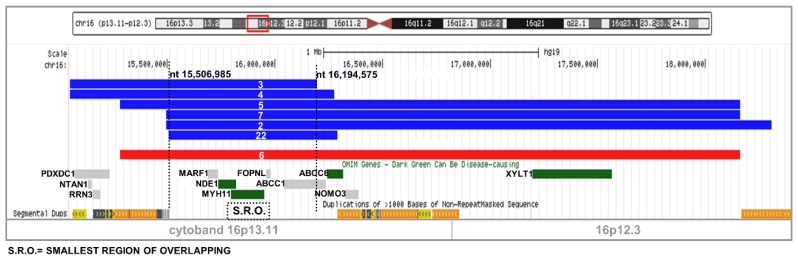
Smallest region of overlapping on 16p13.11. One bar corresponds to one patient and the number refers to the patients reported in Table 1. Red bar indicates a deletion, blue bar a duplication. OMIM genes and segmental duplications are reported.

Seven patients (four males and three females) were found with a 16p13.11 rearrangement: six were duplications and one a deletion (see Table 1 and Figure 3). CNV of patients 2, 3, 4, and 7 were maternally inherited, while the duplication of patient 5 was paternally transmitted; for two cases (cases 6 and 22) parental samples were not available. The shared region spans over 3.2 Mb, while the SRO is 687 kb, encompassing four online mendelian inheritance in man (OMIM) genes (*MARF1*, *NDE1*, *MYH11*, *FOPNL*) and one gene is partially included (*ABCC1*). Interestingly, a proximal larger region that includes *ABCC1*, *ABCC6*, *NOMO3*, and *XYLT1* was shared by four of the seven cases. The boundaries of these two regions of overlapping combine with three blocks of segmental duplications (Figure 3), assuming these rearrangements were derived by the NAHR-mechanism.

### 2.2. Recurrent Microdeletion/Duplication of 16p12.2

**Figure 4:**
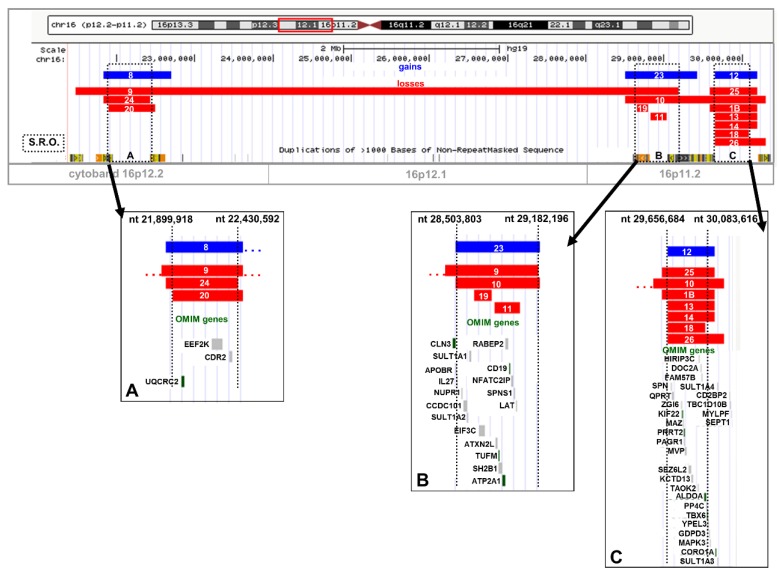
(**A**) 16p12.2 smallest region of overlapping (**B**) 16p11.2 distal smallest region of overlapping (**C**) 16p11.2 proximal smallest region of overlapping. One bar corresponds to one patient and the number refers to the patients reported in Table 1. Red bar indicates a deletion, blue bar a duplication. OMIM genes and segmental duplications are reported.

We found a microduplication in patient 8 and three microdeletions in patients 9, 20, and 24 (Table 1 and Figure 4A). The 16p12.2 SRO spans over 530 kb and three OMIM genes were included (*UQCRC2, EEF2K,* and *CDR2*). Two CNVs were paternally inherited (patients 20 and 24). The patient 24 was a prenatal diagnosis under the indication of intra-uterine growth retardation (IUGR) and oligohydramnios. Lastly, the microdeletion of patient 9, encompassing 7.7 Mb, was included in the distal SRO at 16p11.2 with 70 other missing genes. Again, all the boundaries of the breakpoint regions overlap blocks of segmental duplications, supporting NAHR-derived mechanisms.

### 2.3. Recurrent Microdeletion/Duplication of 16p11.2

We found two very close SRO regions at 16p11.2, covering 1.5 Mb and separated by 474 Kb, including twelve patients (nine males and three females) (Figure 4B,C). One microduplication and four microdeletions were found in the distal SRO (Figure 4B), which spans over 678 Kb including nine OMIM genes (*ATXN2L, TUFM, SH2B1, ATP2A1, RABEP2, CD19, NFATC2IP, SPNS1,* and *LAT*). The microduplication was assessed in a prenatal diagnosis of a twin pregnancy and was maternally inherited (case 23). The microdeletion of patient 19 was inherited from the mother, while the microdeletions of patients 10 and 11 were de-novo. In the latter case, the maternal origin was inferred through the analysis of the remaining parental single nucleotide polymorphism (SNP) allele. The microdeletion of patient 10 also covers the proximal SRO. We found one microduplication and seven microdeletions in the proximal SRO which spans ~427 Kb, with at least eight OMIM genes (*CDIPT, SEZ6L2, KCTD13, PPP4C, YPEL3, CORO1A, KIF22, PRRT2*) (Table 1 and Figure 4C). The microduplication of patient 12 was maternally inherited. We assessed that all the microdeletions were de-novo, except for patients 14 and 26, because parental samples were not available. The maternal origin of patient 1B’s de-novo microdeletion was inferred by comparison between parental and proband SNP calls. All the boundaries of breakpoint regions overlap blocks of segmental duplications, supporting NAHR-derived mechanisms.

### 2.4. Atypical Rearrangements of Chromosome 16

**Figure 5:**
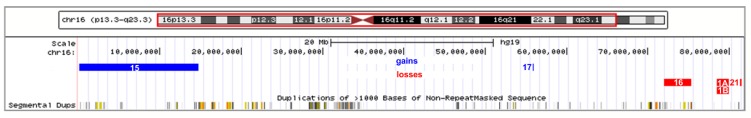
Atypical rearrangements of chromosome 16 associated to clinical signs. One bar corresponds to one patient and the number refers to the patients reported in Table 1. Red bar indicates a deletion, blue bar a duplication. Segmental duplications are reported.

We found five atypical rearrangements of chromosome 16 in two males and four females (Table 1 and Figure 5). In particular, in patient 15 we found a de-novo 14.6 Mb microduplication at 16p13.1–16p13.3 with 48% of mosaicism (see material and methods and Supplementary Figure S1). As the conventional karyotype was apparently normal, we deepened the analysis through the fluorescent in situ hybridization (FISH) method. We found in three out of 100 analyzed cells, an imbalanced translocation as the cause of the partial triplication of the region 16p13.1–16p13.3 on the p arm of chromosome 17, which does not present any loss (Figure 2B). The patient presented a very complex phenotype with growth delay, microcephaly, severe intellectual disability, spasticity, epilepsy, stereotypies, and hepatosplenomegaly, compatible with a mosaic partial gain of over 70 genes, 27 of which are OMIM genes. In patient 17 a de-novo microduplication of 22.5 Kb was assessed at 16q12.2. In patient 16 a microdeletion was seen at 16q22.2–23.1, but the origin could not be ascertained due to lack of samples from the parents. Finally, we found in 16q23.1–q23.2 a familial 1.43 Mb microdeletion in patients 1A and 1B, nephew and paternal aunt, respectively. In both cases, the microdeletion was paternally inherited. Finally, in patient 21 we found a de-novo microdeletion of 200 Kb at 16q23.2.

### 2.5. Additional Variants

Girirajan et al. [18] recently reported an elevated frequency of second-site genomic alterations among patients with severe developmental delay who carry the 16p12.1 microdeletion. As defined by Girirajan’s criteria, a second-site CNV exceeded 500 Kb and mapped to a genomic location that differed from that of the first-site CNV. In our work, we found additional CNVs at a second site in seven out of 26 patients (~27%) (Table 1). We found five cases that fully meet Girirajan’s criteria, while in two patients the second hit was a smaller variant (see Supplementary Table S2). Interestingly, we found a 3.8 Mb deletion in 5q34 that co-segregates with a deletion of 1.4 Mb in 16q23 in a family. These two alterations were both transmitted by apparently healthy parents. In particular, the patient 1B presented a severe phenotype with psychomotor/development delay, bilateral cataracts, and syndactyly, while her brother’s son (patient 1A) had several major and minor malformations, but at the moment we have no data on the psychomotor development. Furthermore, in patient 1B, another additional CNV was observed, a 16p11.2 de-novo deletion of 598 Kb. By SNP genotyping we assessed that this de-novo event arose on the maternal chromosome 16, while the paternal one inherited the familial deletion.

## 3. Discussion

In this study we confirmed the important role of chromosome 16 in determining a pathological phenotype demonstrated through the high density of pathogenic CNVs in our patient series. For the first time, to our knowledge, we considered the entire chromosome 16. We described 26 patients carrying a total of 27 rearrangements (16 microdeletions and 11 microduplications) found during diagnostic investigation of three prenatal and 23 postnatal cases. The association of two recurrent CNVs (16p13.11 and 16p11.2) with increased risk for NDs is well known in the literature [5]. We confirmed that the common denominator ND is present in all postnatal cases to different degrees. Furthermore, as we found CNVs in 14 affected males versus six affected females; we supported the suspicion for a male-biased autosomal effect [23]; this trend is also valid considering only de-novo events (6 males versus 3 females).

In our prenatal cases we identified a 16p13.11 duplication in a 22-week fetus with non-immune hydrops fetalis and dismorphisms (case 22) and a 16p11.2 duplication in an 18-week pair of twins with left ventricular hypoplasia (case 23). Both cases seem to agree with the data of prenatal patients with sSMC(16) (http://ssmc-tl.com/chromosome-16.html) that show partially overlapping duplications, although sSMCs affect much larger regions. In particular, 16-O-p11.2/2-1 showed slowing of growth in the last weeks of pregnancy, while 16-Op11.2/3-1 presented with hygroma colli. In addition, two post-natal cases with sSMC(16) showed a phenotype similar to our cases with regards to speech delay (16-O-p11.1/1-7 and 16-W-p11.2/3-3).

Interestingly, we reported two CNVs in a 21-week fetus with IUGR and oligohydramnios (case 24): a 16p12.2 paternally inherited deletion and a 22q11.23 maternally derived duplication. The coexistence of the two variants could cause a more severe phenotype due to an additive effect (see below).

We were able to analyze the inheritance pattern in 21 patients and it was possible to infer a de-novo origin for nine CNVs, while 12 CNVs were inherited from an apparently healthy parent (six maternal and six paternal). Unfortunately, it was not possible to confirm the parental bias of de-novo pathogenic CNVs pointed out by Ma et al. [24] because the SNP analysis was informative only for two of the nine de-novo cases.

It was reported that deletions have higher penetrance than duplications [7]. We found that five out of twelve inherited CNVs were deletions, while seven were duplications. Despite the numbers here presented being very low, our data seem to confirm that duplications are often associated with benign features and therefore show a penetrance defect. Indeed, considering only the cases in which the parental origin could be identified, the penetrance for duplication events was 56% while for deletion events it was 70%. Conversely, in our nine de-novo series, seven were deletions, while two were duplications; therefore, deletions are more frequently de-novo events than duplications: 7:12 (58%) versus 2:9 (22%).

### 3.1. Dissection of the Chromosome 16 CNV-Morbidity Map

Six out of eleven microduplications were clustered in 16p13.11, and five out of six were inherited from apparently normal parents (Figure 2). As reported elsewhere [20,25,26,27], recurrent duplications in 16p13.11 are present in equal frequency in the normal and the patient population, indicating that this variant is compatible with a normal phenotype. In this work we did not find the typical recurrent 1.65 Mb reciprocal deletion/duplication [20], but we found a common smallest region of overlapping which covers nearly 0.7 Mb and includes the seven rearrangements reported in this region. In addition, by searching for ohnologs in 16p13.11 we found the same five genes already reported [23], three of which are included in the SRO (*NDE1, MYH11,* and *ABCC1*), suggesting these five genes are strong candidate genes for the observed pathological states. In particular, the *MYH11* represents the most important candidate for the predisposition to Thoracic Aortic Aneurysm and Dissection [21], while the *NDE1* gene represents the strongest candidate for the neurodevelopmental phenotypes. Regarding the phenotype correlation, we observed aortic defects in two patients (2 and 7). As a matter of fact, although the classical recurrent 16p13.11 duplication may be a benign variant, it is striking that our duplication carriers not only presented intellectual disability, but also may have severe language delay (patient 3) and behavioral problems (patients 2 and 3). Interestingly, this last clinical sign was also reported in other members of families with duplications [20,25], hence, it seems plausible that 16p13.11 duplication carriers may have a predisposition for aggressive behavior.

Multiple recurrent de novo events, including deletions of 16p11.2, have been strongly associated with autism [28] and other psychiatric and developmental disorders [29]; in this work, we confirmed this specific association because six out of nine de-novo events were 16p11.2 microdeletions.

Recently, the phenotype of the ~ 550 kb 16p11.2 microdeletion syndrome was extended to include a rare, severe, and persistent Childhood Apraxia of Speech [19,30,31]. Curiously, in our series of ten patients with 16p11.2 microdeletions we identified a male bias for this specific phenotype: four male cases, (11, 19, 13, 18) with deletion at 16p11.2 showed dyspraxia. Moreover, other difficulties of speech and language delay were registered in three additional male cases (9, 10, 25). Regarding the remaining three cases with 16p11.2 microdeletions: two were females (1B and 14) and one was male (26), but these clinical signs have not been evaluated yet because he is still too young.

Searching for ohnologs in this region found 24 genes that may have an important role in the development of pathological states. However, the huge number of genes in this region makes it difficult, at the moment, to identify the best candidate for the described phenotype.

As regards the atypical rearrangements of chromosome 16, three out of five were de-novo events (one was in mosaic form, hence, it is a post-zygotic error). It is always very difficult to make a phenotype correlation in this type of non-recurrent rearrangement because different genes and different size/type of CNVs are involved. In two patients, 1A and 16, we found axial hypotonia. In the latter we found EEG anomalies, while in patient 15 we found neurological deficits and stereotypies.

### 3.2. Supporting the Second-Site Genomic Alterations to Explain Incomplete Penetrance and Variable Expressivity

Our study strongly supports the two-hit model for severe developmental delay and for the variable expressivity in microdeletion/microduplication syndromes. As a matter of fact, we observed a second-site genomic alteration in seven out of 26 patients (~27%), five out of 16 considering only microdeletions (31%), in accordance with the literature [23]. Others reported on a particular association between 16p13.11 or 16p12.1 and a second-site genomic alterations [23,32]; we found a specific enrichment between CNVs at 16p12.2 and 16p11.2 (four cases of seven) and a second-hit in another genomic region. Considering the first hit on chromosome 16, we found three paternally inherited microdeletions, two de novo microdeletions, one de novo duplication, and one maternally derived duplication (Supplementary Table S2). Different from the literature [22], in our study the second hit was inherited in 100% of patients: in two cases from the same parent of the first hit; in two cases from the other parent with respect to the first hit; in three cases the first hit was a de novo event. We confirmed a significant bias toward maternal transmission of second-site variants, since we found five cases out of seven cases were maternally derived [22,33]. In addition, in patient 1B we found a third hit: a de novo loss on 16p11.2, which we believe to be the major determinant of the phenotype.

In conclusion, we strongly support the two-hit model for an additive effect of the two independent genomic variants to convert a near-healthy phenotype to a compromised phenotype with extended variable expressivity. The role of CNVs of chromosome 16 is increasingly clear on the determination of developmental delay, considering the high frequency of CNVs we found in our patients. We also found that in some cases (27–31%) a second-site CNV could explain the phenotypic heterogeneity by a simple additive effect or a pejorative synergistic effect, if it affects the same pathway of the first hit. Such epistatic effects could also derive from a third hit, still to be discovered, perhaps as a point mutation or an epigenetic mutation, which could give an interpretation in those cases where both events are inherited from the same apparently healthy parent, and it could also explain penetrance defects.

## 4. Materials and Methods

### 4.1. Participants

We studied 1476 patients (366 prenatal and 1110 postnatal diagnosis); the most frequent indications among postnatal cases were unexplained mental retardation and/or multiple congenital anomalies (MR/MRC). A summary of the phenotypic characteristics of the 27 cases is reported in Table 1. Informed consent was collected for all patients. The study was conducted following the normal diagnostic procedures of the Clinical Pathology Department, S. Gerardo Hospital, Monza, Italy. Participants were enrolled after written informed consent was obtained from parents or legal guardians.

### 4.2. Cytogenetic and FISH Analysis

Peripheral blood metaphases were obtained from phytohaemagglutinin-stimulated lymphocytes, cultured with Chromosome Kit & Medium P (Euroclone, Milano, Italy) according to manufacturer’s protocol. Chromosome analysis was carried out by applying QFQ banding (Q-Bands by Fluorescence using Quinacrine) according to routine procedures, and karyotypes were reconstructed following the guidelines of ISCN 2016 (International System for Human Cytogenomic Nomenclature 2016, Karger).

Fluorescent in situ hybridization (FISH) analysis was carried out according to the manufacturer’s protocol for 16p13.3 (CREBBP gene) and 16p13.13 (TNFRSF17 gene) SureFISH probes (Agilent Technologies, Santa Clara, CA, USA).

### 4.3. Array CGH Analysis

Genomic DNA from peripheral blood samples from patients and their parents was extracted using Wizard Genomic DNA Purification Kit (PromegaTM, Mannheim, Germany) according to the manufacturer’s instructions. DNA concentration was determined on a NanoDrop ND-1000 spectrophotometer (NanoDrop Technologies, Berlin, Germany).

Genomic copy number analysis was performed by array-CGH using an Agilent Human Genome CGH+SNP Microarray 4×180K kit (Sure Print G3, ISCA, International Standards for Cytogenomic Arrays-format, Agilent Technologies, Santa Clara, CA, USA) that includes 110,112 CGH 60-mer probes with 25.3 Kb overall median probe spacing (5 Kb for ISCA regions) and 59,647 SNP probes that enable genotyping and detection of LOH (loss of heterozygosity) with median resolution of 8 Mb. One patient was analyzed by Agilent Human Genome CGH microarray 244 K kit (Agilent Technologies) that includes 236,381 CGH 60-mer probes with 8.9 Kb overall median probe spacing (7,4 Kb in RefSeq genes). Array-CGH experiments were performed following manufacturer’s recommendations, with sex-matched reference DNA. The reference DNA is a high quality, genotyped DNA from normal male and female individuals, Caucasian ethnicity included in Agilent genomic labeling kit.

Analysis was performed using Feature Extraction v10.7 and DNA Analytics v7.0 software (Agilent Technologies) or using Agilent CytoGenomics Software v3.0. Copy number variation profiles provided by the two software programs were comparable. The ADM2 algorithm was applied for both the software programs with a threshold of 5, the putative chromosome copy number changes were defined by intervals of three or more adjacent probes (five or more for prenatal diagnosis samples) and considered as duplicated or deleted when the results exceeded the minimum absolute average log2 ratio that depends on an estimated DLRS value (Derivative Log Ratio Spread), a quality control parameter which differs among all samples. In particular, non-mosaic gain and loss are identified by standard log2 ratio values for all samples: values above 0.6, corresponding to three copies, identify non-mosaic gains; values below −1, corresponding to 1 copy, identify non-mosaic losses. Accordingly, log2 ratio values ranging between the DLRS value and 0.6 refer to mosaic gains, while log2 ratio values between the DLRS value and −1 refer to mosaic losses; the percentages of mosaicism were calculated by the formula reported by Cheung, S.W. et al. [34].

SNP genotyping of de-novo CNVs enabled the identification of parental origin as children’s alleles were inferred by comparison between parental and proband SNP calls. All nucleotide positions were referred to the Human Reference Sequence (GRCh37) Assembly Feb. 2009 hg19 of UCSC Genome Browser (http://genome.ucsc.edu/). Molecular karyotypes were expressed according to ISCN 2016 (International System for Human Cytogenomic Nomenclature 2016, Karger).

The identified CNVs were classified according to previously published criteria [35,36] and by the comparison with the following international databases: Database of Genomic Variants (DGV) (http://projects.tcag.ca/variation) to assess polymorphic variants; the Children’s Hospital of Philadelphia (CHOP) Database (http://cnv.chop.edu/) that includes CNVs found in healthy children; the Database of Human CNVs hosted by IRCCS Oasi Maria SS of Troina (http://gvarianti.homelinux.net/gvariantib37/index.php) that is an Italian Database for CNV from Italian children with mental retardation and disability; the ISCA (International Standards for Cytogenomic Arrays) Consortium Database (http://dbsearch.clinicalgenome.org/); and DECIPHER (Database of Chromosomal Imbalance and Phenotype in Humans using Ensemble Resources; https://decipher.sanger.ac.uk/). Briefly, a CNV was classified as pathogenic when well documented in the literature and if it overlaps a genomic imbalance in a CNV Database of patients with intellectual/developmental disabilities, autism spectrum disorders, or multiple congenital anomalies. A CNV was classified as likely to be pathogenic when it is reported in few/single affected individuals with a similar phenotype or the CNV overlaps only partially with known region involved in micro-deletion/duplication syndromes, or the CNV contains dosage-sensitive genes associated with pathology. A CNV was classified as benign when it is described in the DGV Database and is observed in >1% of apparently normal individuals. A CNV was classified as likely benign when it is not listed in the databases and it has been inherited from an apparently normal parent, or it does not contain genes, or it is found in intronic regions, or when the CNV has been observed in a small number of apparently normal individuals and it is not a common polymorphic CNV. A CNV was classified as uncertain when it has never been observed, when it contains a single gene or genes with unknown function, or a causative gene of a specific pathology not observed in the patient.

### 4.4. Evolutionary Genetic Analysis

Ohnologs were defined as described by Makino and McLysaght [37]. Ohnologs are syntenic genes located on paralogous chromosomal regions and derived from whole genome duplication (WGD). We searched the database http://ohnologs.curie.fr/cgi-bin/SearchPage.cgi for the genes in the regions 16p13.11, 16p12.2, and 16p11.2.

## Figures and Tables

**Table 1:** Patient information.

Case (n)	Sex/Age (y/m/w/d)	GD/PCTL	ID and/or LD	D	Major and Minor Malformations	Heart Defects	CNS Anomalies	Other	CNV on 16 Second Hit	Size (Mb) (SRO Figure 4)	Origin
22 AF	M, 22w+2d	-	-	+	-	-	-	Non−immune hydrops fetalis	16p13.11 dup	0.76	?
3	M, 11y	−	Moderate ID Severe LD	+	−	−	Syncopal episodes with spasticity NE−EEG abnormalities	Aggressiveness hypermetropy	16p13.11dup	1.15	mat
4	F, 4y	−	−	−	−	−	Epilepsy	-	16p13.11 dup	1.3	mat
5	F, 7y	−	Mild ID	+	MI Clinodactily Absence of the 12^th^ rib	-	Abnormal signal intensity of frontal white matter	-	16p13.11 dup	2.88	pat
2	M, 22y	−	ID	+	-	Bicuspid aorta	-	Cryptorchidism Aggressiveness Hypertrichosis	16p13.11–p12.3 dup 15q11.1q11.2 del	2.8 2.7	mat mat
7	F, 16m	+	−	+	rMA	Tricuspid insufficiency	Hypertonia	Silver Russel like phenotype	16p13.11–p12.3 dup	2.7	mat
6	M, 38y	−	ID	−	Hip dysplasia Pectus carinatum	−	−	-	16p13.11–p12.3 del	2.9	?
24 AF	F, 21w+1d	IUGR		-	−	−	−	Oligohydramnios	16p12.2 del 22q11.23 dup	0.59 A 1.29	pat mat
20	F, 3m	−	-	−	Preaxial polydactyly of the hand	2 VSD, PFO, SH	−	Ectopic kidney	16p12.2 del	0.59 A	pat
8	M, 8y	−	ID and LD	+	−	−	Hypotonia Areflexia	Conductive hearing loss	16p12.2 dup	0.86 A	pat
9	M, 24y	+	Absent speech	+	Bilateral clubfoot PUV Bifid uvula	-	Hypotonia	Behavioural problem Hypomobility of palate	16p12.2–p11.2 del	7.7 A−B	?
11	M, 10y	97th	MLD Executive dyspraxia	+	Brachydactyly, Mild flat feet Dolichocephaly	-	EEG abnormalities	Ascending testis	16p11.2 del 3p26.3 dup	0.21 B 0.36	de novo pat
19	M, 10y	3rd	Mild ID MLD Dyspraxia	+	Genu valgum Extra rotation feet	-	-	Strabism Gynecomasty Hypogenitalism	16p11.2 del	0.14 B	mat
23 AF twins	F, 18w+1d	-		-	-	Left ventricular hypoplasia	-	-	16p11.2 dup	0.92 B	mat
	F, 18w+1d	-		-	-	-	-	-	16p11.2 dup	0.92 B	mat
10	M, 4y	−	Mild PDD Severe LD	−	Metatarsus adductus	ASD FPO	Hypertonia	Aggressiveness Hypermetropy	16p11.2 del	1.8 B–C	de novo
13	M, 13y	−	Mild ID Severe LD Verbal dyspraxia	+	Genu valgum Flat feet	−	Chiari malformation type 1	Hepatic steatosis Scoliosis	16p11.2 del	0.53 C	de novo
14	F, 8y	97th	Mild ID	+	−	−	Epilepsy Periventricular nodular heterotopia	Lumbar hyperlordosis Isolated premature thelarche	16p11.2 del	0.53 C	?
18	M, 7y	−	LD Dyspraxia	+	−	−	−	Scoliosis	16p11.2 del	0.43 C	de novo
1B*	F, 32y	5th	PDD	−	Syndactyly	−	−	Bilateral cataract	16p11.2 del 16q23.1–q23.2 del 5q34 del	0.59 C 1.425 3.811	de novo pat mat
25	M, 13y	−	Mild ID LD	+	MA Metatarsus adductus Feet overpronation Crossover toe	−	−	Conductive hypoacusia Scoliosis adenoid hypertrophy Hypermetropy Astigmatism Precocious puberty Obesity	16p11.2 del 22q11.21–q11.22 dup	0.59 C 1.42	de novo mat
26	M, 2y	−	PDD	−	−	−	Hypotonia hyporeflexia	Hyperkeratosis	16p11.2 del	0.65 C	?
12	M, 12y	+	Severe ID Severe LD	+	−	−	Cerebellar hypoplasia	Cornelia De Lange-like phenotype	16p11.2 dup	0.54 C	mat
15	F, 33y	+	Severe ID Severe LD	+	MI	−	Spasticity Epilepsy	Pervasive developmental disorder	16p13.1–16p13.3 gain +	14.6	de novo
1A	M, 2y	−	-	+	Clubfoot Hip delay maturation	−	Axial hypotonia Lower limb hypertonia Hyporeflexia	Bilateral cryptorchidism	16q23.1–q23.2 del 5q34 del	1.425 3.811	pat pat
16	F, 15m	+	Mild PDD	+	Plagiocephaly	−	Axial hypotonia	Ptosis GE reflux	16q22.2–23.1 del	3.35	?
17	F, 23y	+	DD	+	−	−	Mood disorder	-	16q12.2 dup 8q12.1 dup	0.022 0.025	de novo mat
21	M, 10y	−	Mild LD	−	−	−	−	−	16q23.2 del	0.206	de novo

**(y/m/w/d): year/month/week/day;** AF: amniotic fluid; ASD: Atrial Septal Defect; CNS: central nervous system; D: Dismorphisms; DD: Development Delay; GD: Growth delay; GE:gastroesophageal; ID: Intellectual disability; IUGR: Intra-Uterine Growth Retardation; LD: language delay; MA: Macrocephaly; MI: Microcephaly; NE-EEG: non-epileptiform EEG abnormalities; PCTL: percentile; PDD: Psychomotor/Development Delay; PFO: patent foramen ovale; PUV: Posterior urethral valve; disorder; r: relative; SH: Septal hypertrophyMLD: SRO: smallest regions of overlapping; VSD: Ventricular septal defect; Mixed learning disabilities; *1B: paternal aunt of 1A; +: mosaic translocation.

## Supplementary Materials

Supplementary materials can be found at https://www.mdpi.com/1422-0067/20/5/1095/s1.

## Abbreviations

CAs	congenital abnormalities
CAS	childhood apraxia of speech
CGH	comparative genomic hybridization
CNVs	copy number variations
DLRS	derivative log ratio spread
EEG	electroencephalography
FISH	fluorescent in situ hybridization
ID	intellectual disability
ISCN	International System for Human Cytogenomic Nomenclature
IUGR	intrauterine growth restriction
MR/MRC	mental retardation and/or multiple congenital anomalies
NAHR	non-allelic homologous recombination
NDs	neurodevelopmental disorders
OMIM	online mendelian inheritance in man
SNP	single nucleotide polymorphism
SRO	smallest region of overlapping
WGD	whole genome duplication
